# Selenium Biofortification Impacts the Tomato Fruit Metabolome and Transcriptional Profile at Ripening

**DOI:** 10.1021/acs.jafc.3c02031

**Published:** 2023-08-28

**Authors:** Anton Shiriaev, Stefano Brizzolara, Carlo Sorce, Gaia Meoni, Chiara Vergata, Federico Martinelli, Elie Maza, Anis Djari, Julien Pirrello, Beatrice Pezzarossa, Fernando Malorgio, Pietro Tonutti

**Affiliations:** † Crop Science Research Center, Sant’Anna School of Advanced Studies, 56127 Pisa, Italy; ‡ Research Institute on Terrestrial Ecosystems, CNR, 56124 Pisa, Italy; § Department of Biology, University of Pisa, 56126 Pisa, Italy; ∥ Magnetic Resonance Center (CERM) and Department of Chemistry “Ugo Schiff”, University of Florence, 50019 Sesto Fiorentino, Italy; ⊥ Department of Biology, University of Florence, 50122 Florence, Italy; # Laboratoire de Recherche en Sciences Végétales-Génomique et Biotechnologie des Fruits − UMR 5546, Université de Toulouse, CNRS, UPS, Toulouse-INP, 31062 Toulouse, France; ∇ Department of Agriculture, Food and Environment, University of Pisa, 56124 Pisa, Italy

**Keywords:** sodium selenate, selenium nanoparticles, RNA-seq, ripening physiology, hormonal signaling, volatiles, polyphenols

## Abstract

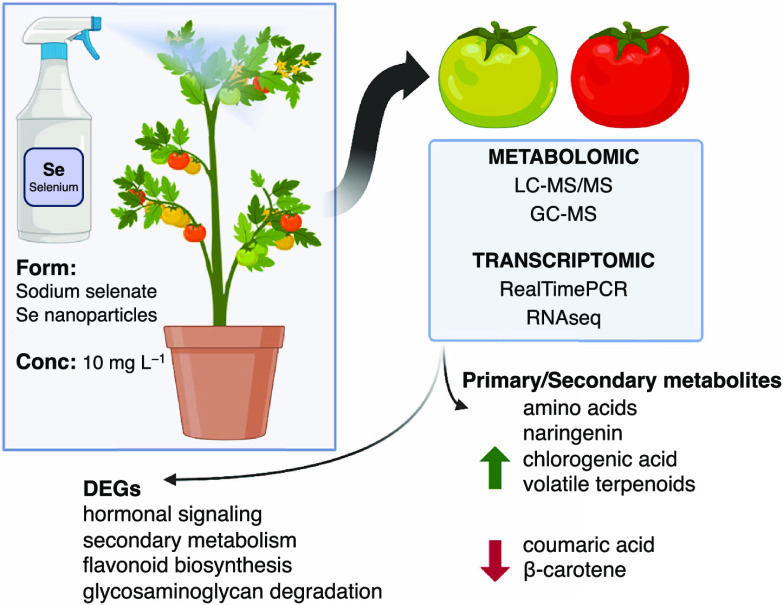

In the present work, the effects of enriching tomatoes with selenium were studied in terms of physiological, metabolic, and molecular processes in the last stages of fruit development, particularly during ripening. A selenium concentration of 10 mg L^–1^ with sodium selenate and selenium nanoparticles was used in the spray treatments on the whole plants. No significant effects of selenium enrichment were detected in terms of ethylene production or color changes in the ripening fruit. However, selenium enrichment had an influence on both the primary and secondary metabolic processes and thus the biochemical composition of ripe tomatoes. Selenium decreased the amount of β-carotene, increased the accumulation of naringenin and chlorogenic acid, and decreased the coumaric acid level. Selenium also affected the volatile organic compound profile, with changes in the level of specific apocarotenoid compounds, such as β-ionone. These metabolomic changes may, to some extent, be due to the impact of selenium treatment on the transcription of genes involved in the metabolism of these compounds. RNA-seq analysis showed that the selenium application mostly impacted the expression of the genes involved in hormonal signaling, secondary metabolism, flavonoid biosynthesis, and glycosaminoglycan degradation.

## 1 Introduction

The biofortification of the edible part of plants with selenium (Se) helps to compensate for a Se deficiency in the human diet, thus reducing the likelihood of certain diseases. After Se is taken up by plants in general as Se(IV) or Se(VI), it is incorporated into Se-amino acids (Se-cysteine and Se-methionine) through the metabolic pathway of sulfur, similar to Se in its chemical properties.^1^ When the concentration of Se accumulated in plants exceeds the optimal levels, it can lead to toxic malformed proteins.^2^ However, although considered as a nonessential element at suitable concentrations, Se has a positive effect on plant metabolism and composition. Such positive effects on plant growth, as well as biochemical and metabolic processes, have been reported particularly in hyperaccumulator species.^3^

Less is known about the possible effects and impact of Se on the metabolic and physiological processes of non-hyperaccumulator species, and, particularly, of specific organs, such as fleshy fruits. Tomatoes are among the most consumed horticultural products worldwide and are recognized as an important source of nutrients and nutraceuticals. Several Se biofortification strategies, including spraying sodium selenate and nanoparticles that transport elemental Se to the entire tomato plant, have been described.^4^

Selenium enrichment tends to delay fruit ripening in tomatoes. Zhu et al.^5^ found a lower production of ethylene and CO_2_ in the tomato fruit grown on plants treated with 1 mg Se L^–1^ by spraying at the beginning of flowering. Pezzarossa et al.^6^ and Puccinelli et al.^7^ observed a general delay in fruit ripening onset and a lower ethylene emission rate in trials, in which Se was supplemented to the nutrient solution of tomato plants at concentrations ranging from 1 to 1.5 mg Se L^–1^. Schiavon et al.^8^ and Castillo-Godina et al.^9^ also reported a generally better fruit performance during the shelf life and storage of Se-enriched fruit.

However, there is limited information available on how Se causes compositional changes and the related metabolic/molecular processes are involved. There is evidence in the literature on the impact of Se on the biochemical composition of fruit, especially secondary metabolites. A delay in carotenoid accumulation^7^ and, specifically, a decrease in β-carotene content^6^ have been detected in Se-enriched tomato fruit at ripening. Nancy and Arulselvi^10^ reported an increase in total phenols, total proteins, nitrates, and a decrease in chlorophyll content in tomato fruit treated with Se when applied twice before flowering at a concentration ranging from 2 to 10 mg L^–1^. Schiavon et al.^8^ showed that Se can enhance the antioxidant activity and increase the phenol content in tomato peel and the amount of naringenin chalcone in fruit flesh. In addition to secondary metabolites, Se supplementation has been shown to impact on several key primary metabolites. For example, when applied at the beginning of flowering at 1 mg L^–1^, Se increased the fruit content of sugars and amino acids.^5^

These effects have been partially explained by the differential expression of individual genes engaged in ethylene production,^5^ the synthesis of carotenoids, and hormonal signaling.^11^ However, the precise mechanism of Se action is still poorly described, and a comprehensive description of the physiological effects of Se enrichment in fruits is still lacking.

In the present work, using transcriptomics and metabolomics analyses, we report the characterization of ripening tomato fruit enriched with Se in terms of physiological and molecular responses, and particularly, the metabolic processes.

## 2 Materials and Methods

### 2.1 Materials and Experimental Design

Tomato plants (*Solanum lycopersicum* L. cv. MicroTom) were grown in a temperature-controlled greenhouse from late August 2019 to January 2020. The cultivation technique, irrigation regime, nutrient solution composition, as well as Se treatment with sodium selenate and Se nanoparticles (SeNPs), the nanoparticle synthesis, and Se determination protocols are those described by Shiriaev et al.^4^

In short, spraying treatment was performed 8 weeks after transplanting, when the most advanced fruit were not exceeding the immature green stage and the least advanced ones were only set after blooming. Plants were treated with 100 mL of a Se solution at a concentration of 10 mg L^–1^ as sodium selenate and SeNPs sprayed on both sides of leaves, flowers, fruit, and stem. On control plants, 100 mL of distilled water was sprayed.

### 2.2 Fruit Selection and Quality Determination

Tomato skin color was used as a marker of ripening evolution, thus ensuring that the sampled fruit were harvested at the same ripening stage. The color was measured with a colorimeter (Konica Minolta CR-10 Plus, Osaka, Japan). Color values were recorded as Hunter *L* and converted into hue angle.

Measurements of ethylene production and color change of the skin have been performed on the fruit collected at the mature green (MG) stage and allowed to ripen off-plant at room temperature (RT). Fruit samples for metabolomic and transcriptomic analyses were collected simultaneously at two ripening stages (MG and red ripe, RR), thanks to the scalar evolution of ripening, 53 days after the treatment. After each sampling, fruit were washed in distilled water. Pericarp tissue including skin was divided from seeds and gel, frozen, and kept at −80 °C in liquid nitrogen.

### 2.3 Tomato Ethylene Production

The production of ethylene was measured at 0, 2, 4, and 7 days after harvest on fruit harvested at the MG stage and allowed to ripen at RT. Each fruit was incubated for 1 h at room temperature in an 80 mL glass jar with a hermetic lid equipped with a PTFE septum. Two 2 mL probes were collected from the headspace of each sample. Ethylene concentration was determined by gas chromatography (HP5890; Hewlett-Packard, Menlo Park, CA) using a flame ionization detector (FID) and a stainless-steel column (150 mm long × 0.4 cm diameter, packed with Hysep T). The temperatures for the GC column and detector were set at 70 and 350 °C, respectively. Nitrogen was used as a carrier gas with a 30 mL min^–1^ flow rate.

### 2.4 Carotenoid Analysis

Three frozen pericarp samples per treatment were analyzed. Each sample was pooled from two biological replicates. About 100 mg FW of frozen tissue sample was pulverized using liquid nitrogen and extracted three times, with 100 μL saturated solution of NaCl + 50 μL of *n*-hexane, then with 200 μL of dichloromethane, and finally with 1000 μL of ethyl acetate. Each extraction ended with centrifugation at 13 200*g* at 4 °C for 5 min. The total organic phase (about 1250 μL) was filtered through a syringe filter (PTFE, 0.45 μm). The remaining water phase was extracted by the same procedure. Eventually, two extracts were pooled.

An aliquot of extract was determined by high-performance liquid chromatography (SpectraSystem instrument, Thermo, Waltham), equipped with a diode-array detector, acquiring spectra between 365 and 650 nm. Samples were loaded on a 250 × 4.6 mm ID C18 Kinetex column (Phenomenex, Torrance), eluted at 1 mL min^–1^ flow rate with the following program: start 100% solvent A for 4 min, next a linear gradient from 0 to 100% solvent B in 10 min, and held at 100% B within 15 min. Solvent A consisted of acetonitrile, methanol, and Tris buffer 0.1 M pH 8 (84:2:14 in volume). Solvent B included methanol and ethyl acetate (68:32 in volume). Quantification of the analytes was performed by reference to a calibration curve. Lycopene and β-carotene content were estimated on the basis of the peaks recorded at wavelengths 472 and 475 nm, respectively.

### 2.5 Selenium Determination

Se determination of the fruit has been described in our previously published paper.^4^ In particular, 0.5 g of each dried powdered tissue sample was processed by microwave-assisted acid digestion with nitric acid and hydrogen peroxide. The total Se content was measured by inductively coupled plasma spectrometry (ICP OES 5900 Agilent, Santa Clara, CA)

### 2.6 Transcriptome Analyses

#### 2.6.1 RNA Extraction and cDNA Synthesis

Four frozen pericarp tissue samples with three biological replicates were grounded in liquid nitrogen with a ceramic pestle and mortar. Total RNA was extracted from one hundred mg of the grounded sample using Spectrum Plant Total RNA Kit (Sigma-Aldrich, Italy). DNA was digested with On-Column Dnase I Digestion Set (Sigma-Aldrich, Italy). The purity and concentration of RNA were tested with a Nanodrop 2000 spectrophotometer (Thermo Scientific, Italy).

A qualitative overview of the RNA sample integrity was performed with a chip electrophoresis assay (Agilent Technologies, Inc). Reverse transcription of RNA to cDNA was carried out using 4 μL of ReadyScript cDNA Synthesis Mix (Sigma-Aldrich, Italy) and 50 ng of RNA. A final 20 μL sample volume was reached by adding RNase free water (Sigma-Aldrich, Italy).

#### 2.6.2 RNA-Seq Data and Differential Expression Analysis

RNA sequencing Library Preparation was performed using NovaSeq 6000 SP Rgt Kit v1.5 (100 cycles) following the workflow of Illumina guidelines. Demultiplexing and data transformation were done by the bcl2fastq module. The basic quality control metrics of the raw sequences were made with FastQC (version 0.11.7), and adapter trimming was performed by TrimGalore (version 0.6.5) script. Mapping to the reference genome (SL4.0 assembly with ITAG4.2 annotation) was done by the Star aligner (version 2.5.1b). Uniquely mapped sequences were filtered using Samtools (version 1.9). A raw count matrix was generated by FeatureCounts software from the Subread package (v.2.0.2). Twelve libraries were sequenced with 3 biological replicates for two ripening stages (MG and RR). From 40 to 55 million reads were generated for each sample and mapped to the ITAG4 tomato genome, annotated with 34688 genes. Each library included from 19500 to 21500 transcripts.

RNA-seq data was validated with quantitative real-time PCR. Sequences of the gene-specific primers (Table S1) were found in the relevant literature or designed using QuantPrime software.^12^ Actin was used as housekeeping genes. Primers were produced by Sigma-Aldrich Merck (Italy). PCR was performed on the CFX Connect Real-Time PCR System (BioRad) using the SYBR Green PCR Master Mix (Life Technologies), reaching the final reaction volume of 10 μL. The PCR conditions were as follows: denaturation at 95 °C for 30 s, 40 amplification cycles with denaturation at 95 °C for 5 s, and annealing and elongation at 60 or 55 °C for 30 s. Following the 40 cycles, a melt cycle was carried out at 65 °C for 5 s and 95 °C for 30 s.

#### 2.6.3 Gene Ontology Enrichment Analysis

Gene ontology and gene enrichment analysis was done utilizing the ShinyGO Web tool (Version 0.741).^13^ Genes and their functions were defined using DAVID Gene Name Batch Viewer^14^ and the annotation developed by Chirinos et al.^15^

### 2.7 Metabolome Profiling

#### 2.7.1 ^1^H NMR

Analysis was performed in CERM/CIRMMP in Florence (Italy). About 100 mg of 5 frozen tomato fruit tissue samples represented by three biological replicates was homogenized with 500 μL of distillate water UltraTurrax (IKA, Germany). After 15 min of centrifugation, 450 μL of supernatant was vortexed with 50 μL of potassium phosphate buffer (1.5 M K_2_HPO_4_, 100% (v/v) 2H_2_O, 2 mM NaN_3_, 5.8 mM TMSP; pH 7.4). 500 μL of the sample was transferred to a 5 mm NMR glass tube and spined on a manual centrifuge.

One-dimensional ^1^H NMR spectra were registered at 400 MHz on an AVANCE III Bruker spectrometer (Bruker, Rheinstetten, Germany) with a 5 mm BBI 400S1 H-BB-D-05Z probe at 300 K. The spectra were obtained with a NOESYpresat (Bruker) pulse sequence, with 128 scans, 33k data point, 12 473 Hz spectral width, 3.3 s acquisition time, 4 s relaxation delay, and 100 ms mixing time.

Phase and baseline distortion in transformed spectra were automatically adjusted and calibrated (TSP peak at 0.00 ppm) with a TopSpin (Bruker). Each 1D spectrum between 0.02 and 10.00 ppm was divided into 0.02 ppm chemical shift bins, and AMIX software (Bruker BioSpin) was used to integrate the corresponding regions. The water section (from 4.95 to 4.7 ppm) was eliminated. Prior to pattern recognition, the data was normalized and the total spectral area was determined for the remaining bins.

Signals were assigned on the spectra with AMIX 7.3.2 (Bruker) matching methods combined with the published literature^16^ and BBIOREFCODE reference database (Version 2-0-0; Bruker). The signals were integrated in the spectra to determine the relative concentrations of each metabolite.

#### 2.7.2 Volatile Organic Compound (VOC) Profiling

VOC analysis has been run employing the protocol reported by Brizzolara et al.^17^ with minor modifications. Briefly, 5 g of pericarp was blended with 5 mL of 1 M NaCl water solution by an UltraTurrax T25 homogenizer (IKA, Germany) to produce tomato purees. Each of 4 samples has been analyzed in triplicate containing two fruits each.

Gas chromatography (Clarus 680, Perkin Elmer, Waltham) together with mass spectrometry (Clarus 600, Perkin Elmer, Waltham) has been used for VOC quantification. 5 g of sample was sealed in a 20 mL crimp vial (Sigma-Aldrich, Italy), incubated within 60 min at 40 °C, and extracted with an SPME fiber (50/30 μm, DVB/CAR/PDMS, Sigma-Aldrich, Italy) for 45 min at 40 °C. GC temperature program was set as follows: 0–1 min, 40 °C; from 40 to 250 °C at the rate 4 °C min^–1^, 1 min on hold; from 250 to 280 °C at the rate 15 °C min^–1^, 1 min on hold. A SLB-5MS column (Fused silica 30 m × 0.25 mm × 0.25 μm film thickness, Sigma-Aldrich, Italy) was utilized for the analysis, and helium at 1 mL min^–1^ constant flow was a carrier gas.

AMDIS software (National Institute of Standards and Technology, Gaithersburg) has been used to identify compounds by comparing the recorded spectra with NIST v. 2 library (National Institute of Standards and Technology) and using retention index (RI) information (standard alkane mix C6-C40, Sigma-Aldrich, Italy). Only compounds with matching levels of 80% or above were considered.

#### 2.7.3 Polyphenol Analysis

The sample extraction and UHPLC-MS/MS analysis protocols and operation source parameters are the same reported by Francini et al.^18^ Briefly, 4 frozen pericarp tissue samples with three biological replicates were grounded under liquid nitrogen, and 100 mg probe was mixed with 2 mL of methanol, filtered with a Whatman cartridge (0.45 μm) and diluted 1:20 with MilliQ water. Targeted quantitative analysis of selected polyphenols has been carried out with a Sciex 5500 QTrap+ mass spectrometer (AB Sciex LLC, Framingham, MA), coupled to a Turbo V ion-spray source and attached to an ExionLC AC System custom built by Shimadzu (Shimadzu Corporation, Kyoto, Japan). Chromatographic separation was made in a Phenomenex Kinetex Biphenyl 2 × 100 mm^2^, 5 μm column (Phenomenex, Torrance, CA) in gradient mode using solvent A (acetonitrile–0.1% formic acid) and solvent B (water–0.1% formic acid) with the following program: 0 min, A 5%; 0–10 min, A 5–95%; 10–12 min, A 95%, followed by 4 min equilibration (A 5%) (300 μL min^–1^ flow rate, 20 μL injection volume, 40 °C column oven temperature). MS/MS analyses were carried out in electrospray negative ion mode with nitrogen as a collision gas.

### 2.8 Statistical Analysis

Metabolomic data were tested using ANOVA with R Studio (2021.09.0+351 Ghost Orchid) considering the Se concentration and ripening stage as explanatory variables. The results were analyzed with the least significant difference (LSD) test (*p* < 0.05). Statistically significant differences between treatments were identified using Student’s *t*-test or Wilcoxon nonparametric test for non-Gaussian data. VOCs, polyphenols, and NMR results were analyzed by partial least squares discriminant analysis (PLS-DA) computed in JMP (v. 16, SAS Institute Inc., Cary, NC).

The results of RT-qPCR were reported as fold change values, calculated by normalizing to the expression level of the reference gene in control samples at the MG stage, and transformed into a logarithmic scale (log_2_^2^ FC). Raw transcript count normalization and differential expression analysis were computed using the DESeq. 2 R package (Version 1.34.0).^19^

## 3 Results

### 3.1 Selenium Content and Fruit Ripening Parameters

**Figure 1:**
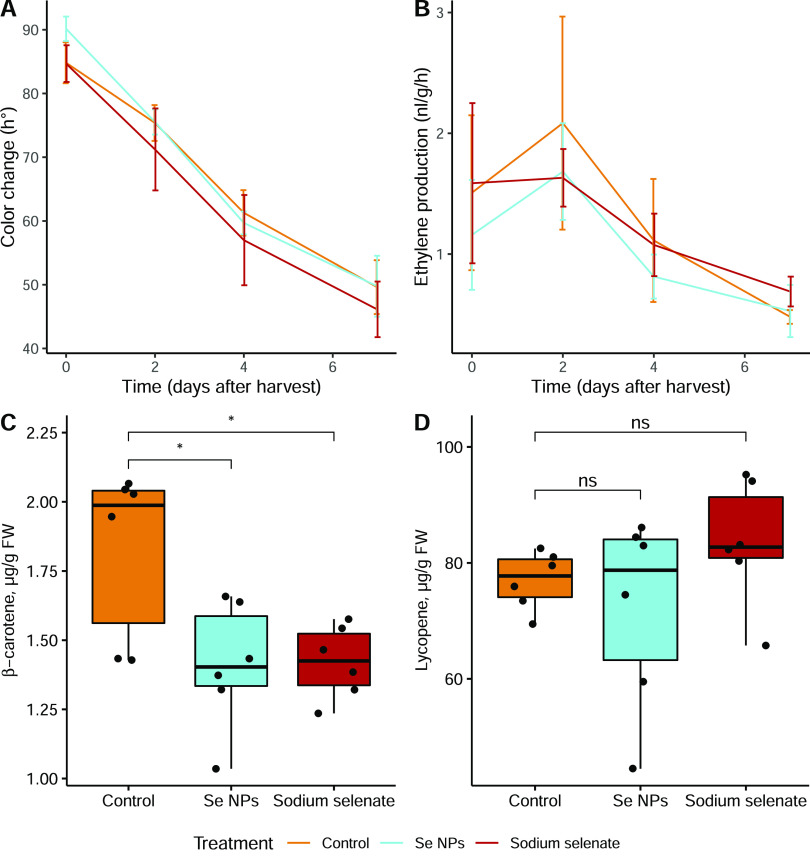
Color (A) and ethylene production (B) measured in intact fruit detached at the mature green (MG) stage and kept at room temperature for 7 days. Panels (C) and (D) report the β-carotene and lycopene content in the RR fruit, respectively. Data are the mean of 4 replicates for color and ethylene and 6 replicates for carotenoid content. A Student’s *t*-test, *p*-value less than 0.05, is flagged with an asterisk (*). The bars indicate the standard deviation.

Treatments with 10 mg L^–1^ sodium selenate and nanoparticles led to a Se accumulation of 1.217 and 0.677 mg kg^–1^ DW in MG tomatoes, respectively, as reported in our previously published paper.^4^ In fruit detached at the MG stage and allowed to ripen off-plant at RT, color evolution, measured by the hue parameter, showed no significant differences compared to the control (Figure 1A), highlighting that the time to ripen did not change in Se-enriched tomatoes.

All fruit, regardless of the treatment, showed a climacteric ethylene peak, with the highest biosynthetic values detected at harvest or after 2 days, followed by a decreasing trend (Figure 1B). No statistically significant differences were detected among the samples.

### 3.2 Carotenoids and Amino Acids

Although the colorimeter data reported no significant difference among samples (Figure 1A), the specific carotenoid analysis showed that the β-carotene content (Figure 1C) was significantly lower than the control in the RR fruit treated with Se. The lycopene content showed no significant differences between treatments (Figure 1D).

**Figure 2:**
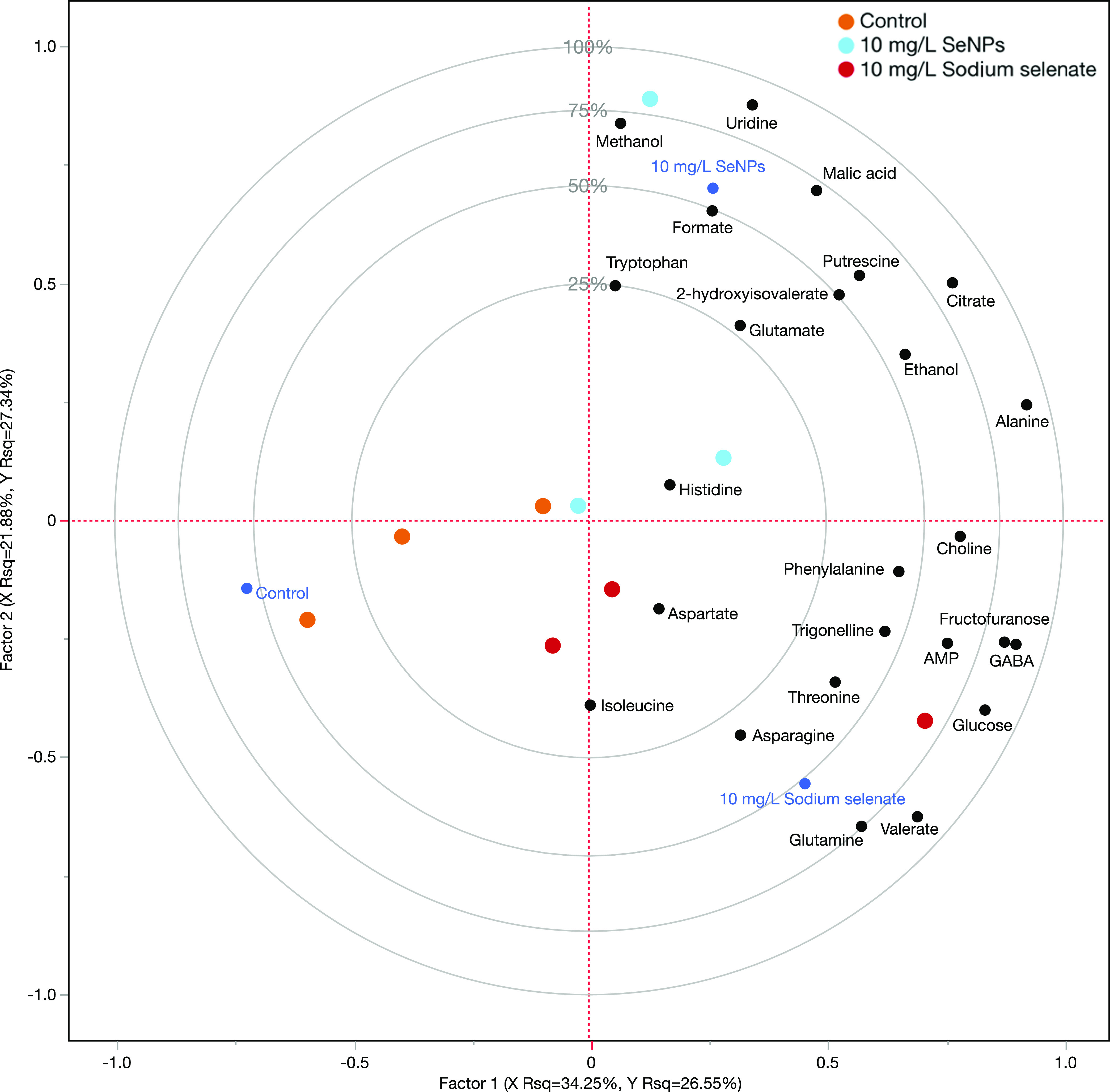
Partial least square discriminant analysis (PLS-DA). The model has been created using the identified compounds detected by NMR-based metabolism analysis of the RR fruit as predictor variables, while Se treatment has been employed as a response variable.

To better evaluate the possible effects of Se enrichment on the composition of the RR fruit, an NMR-based metabolism analysis was then performed. A total of 25 compounds were identified, and the PLS-DA analysis showed that the Se treatment, corresponding to factor 1, had a very limited effect on the primary metabolites and explained only 54% of the total variability within the model (Figure 2). However, samples representing the two treatments locate in different quadrants, which may be the result of different Se concentrations detected. In addition, possible differences could be related to the different chemical form of the Se distributed: Se(IV) and the Se^0^ for the salt and nanoparticle treatments, respectively.

However, the RR fruit with the highest Se concentration (sodium selenate) also had the highest accumulation of amino acids. In particular, γ-aminobutyric acid (GABA) was found to be associated with sodium selenate samples in PLS-DA, and the corresponding VIP score ranking (Table S2). Glutamine, threonine, asparagine, and phenylalanine also appeared to be associated with the salt samples.

### 3.3 Transcriptome Analysis

On the basis of the results obtained in terms of Se concentration and the above reported analyses of RR fruit composition, a transcriptome analysis was performed to identify possible genes involved in the metabolic processes affected by Se in ripening tomato fruit. Control samples and sodium selenate-treated samples, which showed the highest Se concentration, were thus compared. The RNA-seq analysis revealed that a total of 276 and 593 genes were found to be significantly differentially expressed between the Se-treated and control samples at MG and RR stages, respectively.

To verify the results from RNA-seq, the gene expressions with the most significant levels of adjusted *p*-values and noticeable log_2_ fold changes were validated, namely, 1-aminocyclopropane-1-carboxylate oxidase 2 (*ACO*2), NAC domain protein (*NOR*), never ripe-2 (*ETR*3*/NR*), 4-coumarate ligase (4*CL*1), chalcone synthase 1 (*CHS*1), and chalcone-flavanone isomerase (*CHI*3). Their expression pattern under the effect of Se application in RNA-seq was in accordance with RT-qPCR (Figure S1).

Principal component analysis of the 1000 most variable genes revealed that the main contribution to data variation was due to the effect of ripening (PC1, describing 52% of total variance). However, a separation of Se-treated and control samples was observed on PC2, describing 16% of the total variability (Figure S2).

**Figure 3:**
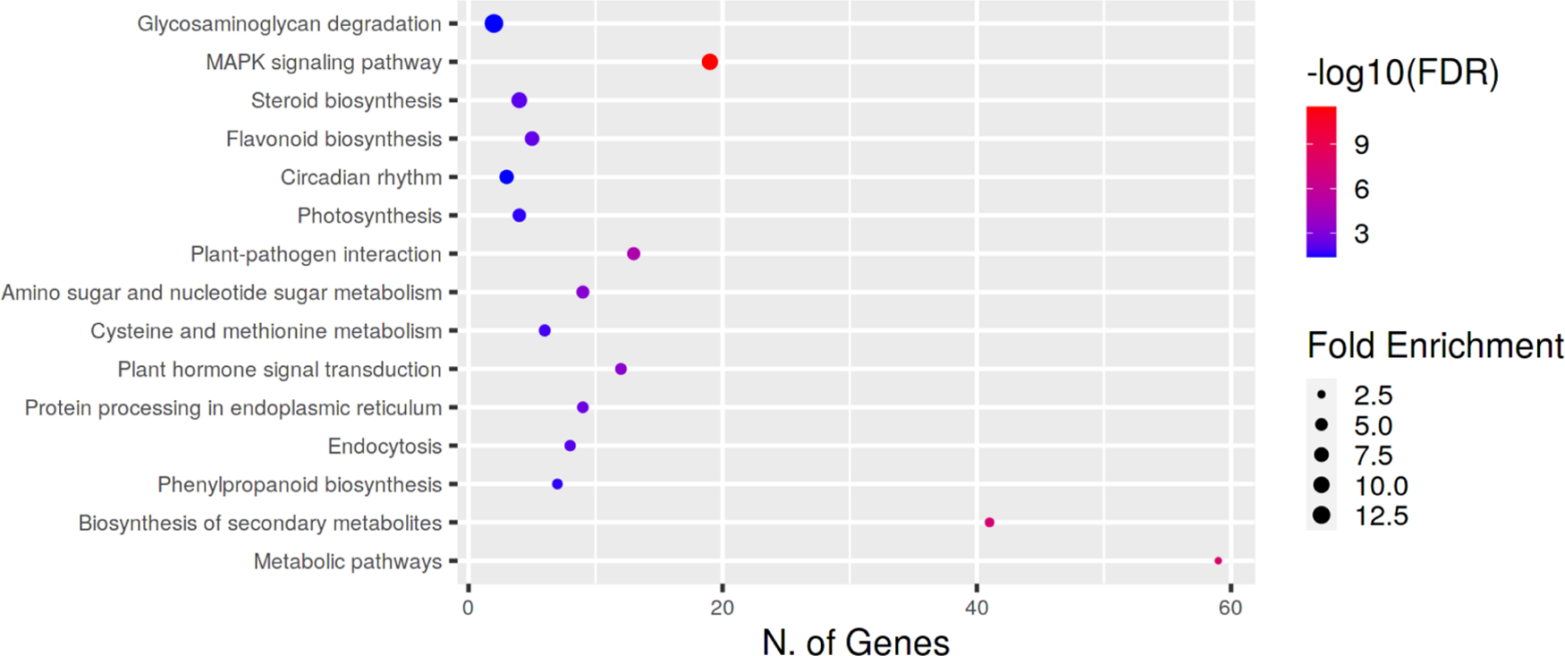
KEGG pathway enrichment analysis of DEGs in Se-enriched tomato fruit. The *Y*-axis of the bubble chart represents the top 15 GO enrichment terms, whereas *X*-axis represents the number of DEGs. Increasing the bubble size indicates an increasing enrichment score. Bubble colors from blue to red indicate an increasing false discovery rate (FDR).

Differentially expressed genes (DEGs) of each ripening stage were extracted and used for gene ontology (GO) enrichment analysis (Figure 3). The biological processes that appeared to be most affected by Se application were the MAPK signaling pathway, plant–pathogen interaction, hormone signaling, and secondary metabolism. However, glycosaminoglycan degradation, flavonoid biosynthesis, and photosynthesis showed the highest enrichment score.

**Figure 4:**
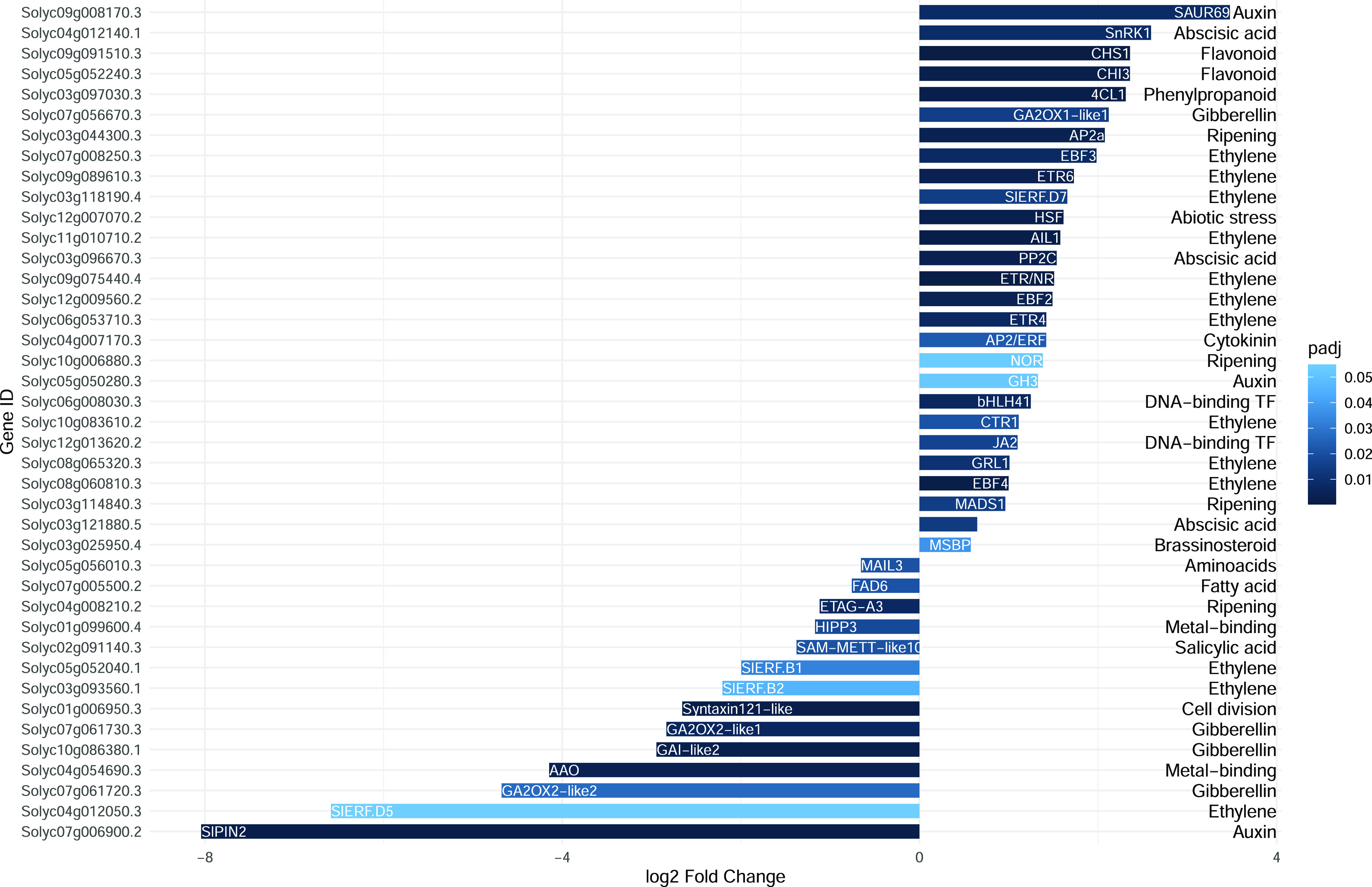
Fold change (log_2_) and functional characterization of DEGs at the MG stage in control and Se-treated fruits. Positive values indicate the genes more expressed in Se-treated fruit and vice versa for negative values. Color intensity indicates the significance level based on the adjusted *p*-value. The descriptions of the DEG names are listed in Table S3. Genes present with no name acronyms were not yet defined according to available annotations; however, their functional category is reported.

**Figure 5:**
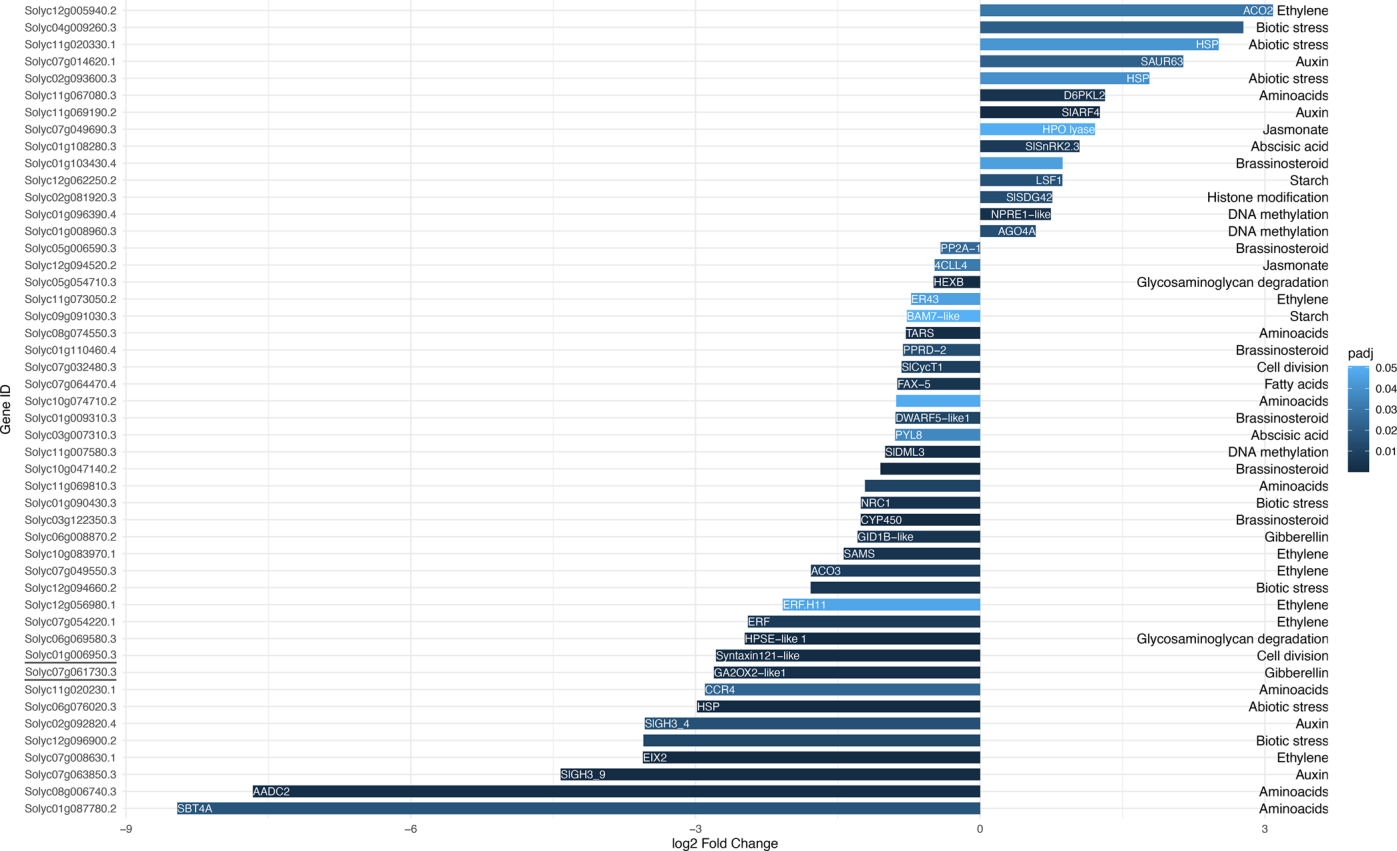
Fold changes (log_2_) and functional characterization of DEGs at the RR stage in control and Se-treated fruit. Positive values indicate the genes more expressed in Se-treated fruit and vice versa for negative values. Color intensity indicates the significance level based on the adjusted *p*-value. The descriptions of the DEG names are listed in Table S4. Genes present with no name acronyms were not yet defined according to available annotations; however, their functional category is reported. The genes expressed differentially during both MG and RR stages are underlined.

To detect those genes that were most impacted by Se, DEGs extracted during the MG and RR stages were manually annotated, and their fold changes were plotted (Figures 4 and 5).

At the transcriptomic level, Se appeared to impact the physiology of different hormone categories, namely, auxins, abscisic acid, gibberellins, brassinosteroids, jasmonic acid, abscisic acid, and ethylene, at both MG and RR stages. In terms of ethylene, GO enrichment analysis of RNA-Seq results revealed a large group of genes involved in its biosynthesis and signaling. Considering the production, *ACO*2 was upregulated at the RR stage, while *ACO*3 was suppressed after the Se treatment. In terms of ethylene perception, *ETR*3 *(NR)*, *ETR*4, and *ETR*6 genes were upregulated at the MG stage. Our results also showed that Se affected the expression of transcription factors *ERFs* (including *ERF.H*11, *ERF.B*1, *ERF.B*2, *ERF.D*7, and *ERF.D*5), which showed both upregulated and downregulated members at MG and RR stages.

Se impacted three genes recognized as ripening-related at the MG stage. Namely, transcription factors ERF/*AP*2, *NOR*, and MADS-box protein 1 (*MADS*1) were upregulated, whereas endoxyloglucan transferase (*ETAG-A*3) was downregulated by Se.

Among the genes affected the most by Se, were also genes involved in epigenetic regulation. At the RR stage, Se upregulated DNA-directed RNA polymerase (*NPRE*1*-like*) and Argonaute 4a (*AGO*4*A*) and downregulated DNA demethylase 3 (*SlDML*3). In addition, Se upregulated the ribulose-1,5 bisphosphate carboxylase/oxygenase large subunit N-methyltransferase (*SlSDG*42) involved in histone modification.

In the RR stage, two genes involved in glycosaminoglycan degradation were suppressed by Se. The heparanase-like protein 1 (*HPSE*) was expressed in Se-treated samples 4.9 times less than in the control. β-Hexosaminidase (β*-Hex*) was suppressed by about 50% in the RR Se-enriched fruit.

Se also impacted a group of genes involved in primary metabolism through amino acid pathways. Namely, serine/threonine-protein kinases (*D*6*PKL*2) were upregulated while threonine-tRNA synthase (*TARS*), serine/threonine-protein kinase-like protein (*CCR*4), aromatic amino acid decarboxylase 2 (*AADC*2), and serine protease (*SBT*4*A*) were downregulated.

Among the genes most affected by Se treatment, some are involved in secondary metabolic pathways, including volatiles and phenylpropanoids. Specifically, genes responsible for consecutive steps of coumarate catabolism were upregulated during the MG stage: 4-coumarate ligase (4*CL*), chalcone synthase (*CHS*), and chalcone isomerase (*CHI*).

### 3.4 VOCs and Polyphenol Profiles in MG and RR Fruit

**Figure 6:**
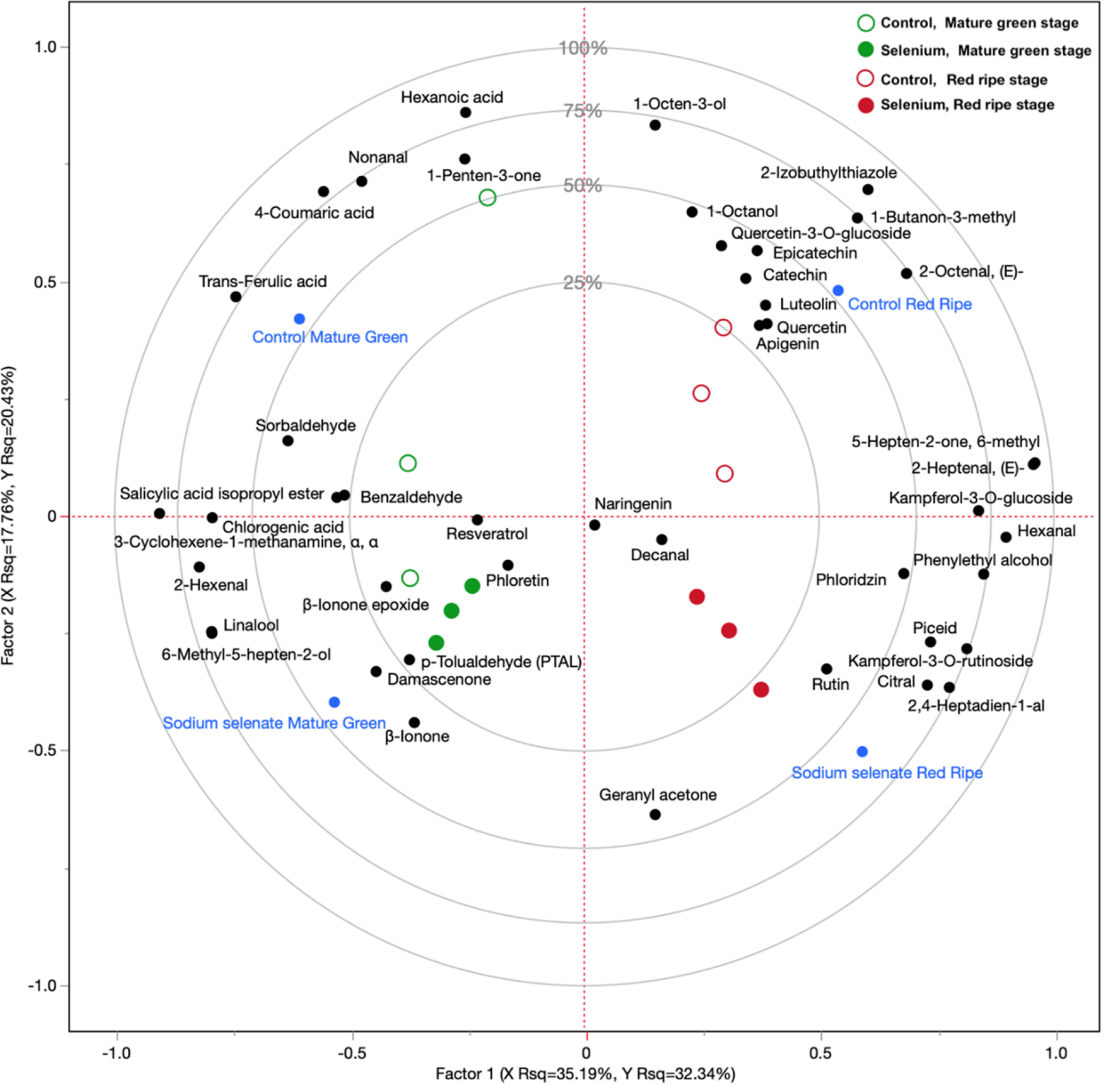
Partial least square discriminant analysis (PLS-DA). The model has been created using the identified VOCs and polyphenols as predictor variables, while a factor combining ripening stage and Se concentration in the fruit has been employed as a response variable. Two ripening stages have been considered: MG and RR stages.

Based on the above described results of RNA-seq regarding genes involved in secondary metabolism, VOC and polyphenol profiling were performed in the control and sodium selenate MG and RR fruit samples. This led to the identification of 29 VOCs (Table S5) and 17 polyphenols (Table S6). Both groups of compounds were shown to be affected in Se-enriched fruit, and a PLS-DA model was created to investigate the Se effects (Figure 6). Taking together the first two factors of the model, approximately 52% of the total variability in the dataset is explained by PLS-DA. Factor 1 mainly describes the effect of ripening, while factor 2 appears to be mainly related to the effect of Se treatment. The ripening evolution is clearly visible in Figure 6 with samples moving from the left to right quadrants, while the effect of Se biofortification is to move samples from the top to the bottom quadrants.

Terpenoids (β-ionone, citral, geranyl acetone, and linalool) appeared to be the most affected chemical class of the VOCs, with a clear increase in Se-treated samples, both during MG and RR stages. Alcohols and aldehydes were also altered by Se treatment. Hexanoic acid, nonanal, and 1-penten-3-one, associated with the aroma of MG fruit, were decreased by Se, while damascenone and *p*-tolualdehyde were increased by the treatment. In addition, 2,4-heptadien-1-al and phenylethyl alcohol were increased by treatment during the RR stage, and 1-octen-3-ol, 1-octanol, 1-butanon-3-methyl, 2-izobuthylthiozole, and 2-octenal (*E*) were, in contrast, more associated with the control sample and RR stage.

Among the identified polyphenols, Se appeared to markedly impact coumarate biosynthesis. Specifically, Se appeared to decrease the production of coumaric acid, while naringenin and phloretin, which are core flavonoid intermediates, appear to be present at higher levels in Se-enriched fruit. At the MG stage, Se decreased the concentration of hexanoic and *trans*-ferulic acids while slightly increasing phloretin. At the RR stage, the PLS-DA model showed that Se affected piceid, flavonol glucosides, as well as rutin and kaempferol-3-O-rutinoside levels, leading to an increase in these compounds. At the same time, epicatechin, catechin, luteolin, quercetin, and apigenin were higher in untreated samples.

## 4 Discussion

The amount of Se detected in the MicroTom fruit in the present trials differed depending on whether salt or nanoparticles were applied, and it was in the range of the nontoxic concentrations reported by other authors in different tomato cultivars.^5−7^ Zhu et al.^5^ reported that ripening was delayed in Se-enriched tomato fruit cv. Provence, and our previous studies observed similar effects in cv. Red Bunch tomatoes.^6,7^ However, in the present experiment, Se-enriched MicroTom tomatoes did not show significant changes in ripening-related parameters, as shown in Figure 1, thus indicating that there may be a genotype-dependent effect.

However, when we analyzed physiological, metabolic, and molecular aspects, there was an effect of Se enrichment in the MicroTom fruit. In fact, the transcript profiles clearly indicate the presence of several hormone-physiology related genes, which were differentially expressed in Se-enriched fruit compared with control.

Although ethylene biosynthesis did not seem to be affected, the upregulation of *ACO*2 and the downregulation of *ACO*3 detected in Se-treated samples possibly indicate the interference of Se in the processes involved in ethylene biosynthesis, particularly in the last step of its production. The suppression of the SAM synthase homolog gene (*SAMS*) during the RR stage may indicate an effect of Se on methionine recycling. In fact, Costa et al.^20^ explained the decrease in ethylene levels in Se-treated cut flowers as being due to a reduction in methionine. Sensitivity to ethylene is controlled by the abundance of ethylene receptors, and ETR3 (NR) and ETR4 appear to play a key role in fruit ripening.^21^ Given that in our study, the expression of *ETR*3 *(NR)* and *ETR*4 was altered in Se-enriched fruit, and Se likely interferes with ethylene sensitivity.

Our KEGG pathway enrichment analysis of DEGs in Se-enriched tomato fruit revealed that categories with the highest number of genes affected by Se are related to hormones and signaling pathways. In particular, we found that several DEGs are involved in auxin signaling and transport (upregulated *SAUR*63, *SAUR*69, *ARF*4, and downregulated *GH*3.4, *GH*3.9, *PIN*2).

Malheiros et al.^22^ also found that auxins had been altered, as in their study, Se treatment downregulated genes involved in auxin signal transduction (*ARF*10, *ARF*19, *GH*3.3, and *GH*3.4), biosynthesis (*YUCCA*1 *and YUCCA*3), and transport (*PIN*1*A*, *PIN*1*B*, and *PIN*3) in rice. Similarly, Dou et al.^23^ reported the suppression of genes responsible for auxin signaling (*Aux/IAA*, *SCF*, *SKP*, and *GH*3) in maize.

As far as we are aware, there are no reports on selenium’s possible impact on gibberellins and brassinosteroids. These two groups of hormones have been abundantly studied in the context of stress responses,^24,25^ suggesting that more detailed future studies on gibberellins and brassinosteroids in Se-enriched plants may provide new information on the possible role of Se regarding stress conditions.

Selenium appears to play a key role in abiotic stress tolerance. Selenium was found to improve the heat tolerance of sorghum (*Sorghum bicolor* L.) and the drought tolerance of spring barley (*Hordeum vulgare* L.)^26^ and sesame (*Sesamum indicum* L.).^27^ Se also reduced the effects of metals and metalloids in various plant species^28^ and increased the ability of tomato (*S. lycopersicum* L.) to withstand NaCl salinity.^29^ Interestingly, our RNA-seq results showed that Se treatment caused the induction of five genes engaged in the regulation of the senescence-related hormone abscisic acid.

At the MG stage, Se induced an upregulation of SNF-related kinase 1 (*SnRK*1), with the second highest fold change among the impacted annotated genes. Wang et al.^30^ found that in tomato, *SnRK*1 upregulation improved salt tolerance by controlling the ABA signaling system and reactive oxygen metabolism.

The upregulated heat shock factor (*HSF*) is known to take part in heat tolerance pathways.^31^ This observation, as well as an upregulation of heat shock protein (*HSP*) genes, may confirm the findings of Malerba and Cerana,^32^ who hypothesized that Se helps in coping with heat stress in plant tissues. Similar results on *HSP* gene expression in relation to Se enrichments have been reported in wheat by Feng and Ma.^33^

Various TFs play key roles in modulating responses to different kinds of stimuli and stresses, of which the *AP*2*/ERF* family are of paramount importance. The ERFs, including *ERF.B*1 and *ERF.B*2, which were downregulated by Se in our dataset, are reported in other studies to be differentially affected by various abiotic stress types in tomato plants.^34^ Taken together, we believe that our transcriptomic data provide new cues on the molecular mechanism underlying the possible role of Se in abiotic stress responses.

The large group of differentially expressed genes that we found to be involved in the plant–pathogen interaction, including mitogen-activated protein kinase (*MAPK*) genes and protein phosphatase 2C (*PP*2*C*), seems to indicate that Se modulates biotic stress as reported by Quiterio-Gutiérrez et al.^35^ Se hyperaccumulators have also been defined as plants with high resistance to pathogen infection since they naturally accumulate Se from the soil.^36^

The downregulation of the threonyl-tRNA synthase (TARS) gene is recognized to slow down the attachment of threonine to tRNA, which may result in higher accumulations of this amino acid in Se-enriched fruit. Higher accumulation of threonine, asparigine, and γ-aminobutyric acid (GABA) in Se-treated tomato plants reinforces the hypothesis that Se serves as a stress elicitor. The GABA shunt, in particular, is involved in the maintenance of the carbon/nitrogen balance, defense from insects, and oxidative stress.^37^ Asparagine accumulation can also be induced by drought, salt, toxic metals, mineral deficiencies, and pathogen attack.^36^ Interestingly, a similar pattern of increasing amino acids appears to characterize tomato fruit under cold storage.^38^

The altered transcriptomic profiles detected in Se-enriched ripening fruit may have a direct or indirect impact on the metabolic pathways and fruit composition, with important consequences on the organoleptic traits and nutraceutical properties.

The lower β-carotene content, confirmed by our previous work^6^ together with the higher β-ionone and damascenone content detected in the Se-enriched fruit of the present experiment, suggests that specific steps of the carotenoid metabolism are affected by Se. This includes both the synthesis and degradation of key molecules, such as β-carotene, which is a precursor of several volatile compounds belonging to the apocarotenoid class. Carotenoids are precursors of apocarotenoids that have key functions as volatiles, signaling molecules, and hormones involved in stress responses.^39^

In terms of volatile apocarotenoids, the Se-induced increase in β-ionone, reported by Tieman et al.,^40^ is interesting as it is one of the compounds related to consumer preferences. Whether the higher production of apocarotenoids, such as β-ionone, β-ionone-epoxide, and geranyl acetone, detected in Se-treated tomato has any relation with the lower concentration of β-carotene detected in the RR fruit, with a beneficial impact on the flavor of Se-enriched tomatoes, still needs verification.

Other compounds influenced by Se treatment could be related to the organoleptic fruit quality. Namely, hexanal and 1-penten-3-one, which are altered by the Se treatment in our analyses, have been described as some of the most odor-active volatiles contributing to the fresh tomato aroma.^41^ Our present results are in accordance with previous findings of our research group on volatilome of tomato fruit enriched with sodium selenate through the root system.^42^

With regard to other processes of the secondary metabolism, three phenol compound biosynthetic genes (4*CL, CHI*3, and *CHS*1) involved in coumaric acid metabolism^43^ were found to be upregulated in the Se-enriched fruit. These results are in line with Behbahani et al.,^44^ who reported that the leaf-soluble phenol content and the expression of the 4*CL* gene were induced in bittermelon (*Momordica charantia* L.) treated with Se nanoparticles. It has also been reported that Se impacted the shikimic acid pathway in cucumber (*Cucumis sativus* L.) seedlings under stress from cadmium toxicity.^45^

A general effect of Se on the phenylpropanoid pathway is also hypothesized considering the changes in coumaric acid, naringenin, phloretin, piceid, rutin, and kampherol-3-O-rutinoside at the final stage of fruit ripening. In particular, the lower production of coumaric acid and abundancy of naringenin and phloretin may be partially explained by the above reported upregulation of flavonoid biosynthesis-related genes 4*CL*, *CHI*3, and *CHS*1. These findings are in line with Schiavon et al.,^8^ who reported that Se was effective in inducing the production of naringenin chalcone and kaempferol and found a decrease in cinnamic acid derivatives. Rutin, kaempferol-3-rutinoside, and naringenin were dominant flavonols in cherry tomato.^46^ Their higher production in Se-enriched tomato fruit could indicate the direct impact of Se on the nutritional value of fruit, while the higher production of stilbenes piceid and resveratrol could possibly be associated with a better response to stress conditions. Enhanced stilbene biosynthesis is considered to improve resistance to salt stress, drought, temperature, and biotic stress, both *in planta* and in plant organ and cell cultures.^47^ Also, transgenic tomatoes with enhanced resveratrol synthesis have been characterized by increased antioxidant activity.^48^

As highlighted above, there is no apparent evidence of a marked effect of the Se concentration used in these trials on the ripening parameters. However, RNA-seq data suggest that several genes involved in the final stages of fruit development are differentially expressed when comparing Se-treated fruit with the control. Besides hormonal and regulatory genes, the altered expression of specific genes suggests that Se may have an impact on late ripening-related events. For example, β-hexosaminidase, downregulated at the RR stage of Se-treated fruit, is involved in N-glycan processing, contributing to ripening-related fruit softening. The β-Hex activity increases with fruit ripening,^49^ and the suppression of N-glycoprotein modifying enzymes in transgenic tomatoes reduced fruit softening, thus enhancing the fruit shelf life, and their upregulation leads to excessive softening.^50^

The differential expression of genes linked to DNA methylation and histone modification may possibly support the hypothesis formulated by Behbahani et al.,^44^ who indicated that the mechanism of Se impact on gene expression could be partly explained by its ability to control certain transcription factors and trigger epigenetic modifications in DNA cytosine methylation and chromatin conformation.

In conclusion, our results show that the application of Se to tomato plants resulted in better nutraceutical status of fruit both directly, by increasing the Se content, and indirectly, by improving the levels of specific beneficial compounds. Se affected the primary and, in particular, the secondary metabolism and changed the accumulation of specific polyphenols, amino acids, terpenoids, and carotenoids in the MicroTom fruit. The VOC profile was potentially improved by increasing the emission of components associated with consumer liking.

To the best of our knowledge, the current study is the first report on the tomato transcriptome following Se biofortification. Our findings provide a partial and hypothetical explanation of the metabolomic changes described above.

To some extent, the observed changes in secondary metabolism may be triggered by the Se impact on the expression of hormone-related genes involved in the physiology of auxins, abscisic acid, and ethylene.

The observed changes in both transcriptional regulation and biochemical composition provide evidence that Se may serve as a stress elicitor in tomatoes due to the increased production of stilbenes, terpenoids, and amino acids characterized in the literature as improving resistance to stress in plants.

## Supporting Information Available

Table S1. Gene name, GenBank number access, forward and reverse sequence of primers used in RT-qPCR for gene expression analyses. Table S2. List of compounds identified with ^1^D NMR analysis, ranked based on VIP-score (variable in projection, weighted sum of the squared correlations between the PLS-DA components and the original variable) according to the PCA analysis described in Figure 3. Figure S1. Expression pattern of flavonoid biosynthetic genes 4-coumarate ligase (4*CL*1), chalcone synthase 1 (*CHS*1), chalcone-flavanone isomerase (*CHI*3), and ethylene-related genes 1-aminocyclopropane-1-carboxylate oxidase 2 (*ACO*2), NAC domain protein (*NOR*), never ripe-2 (*ETR*3|*NR*). qRT-PCR data, showing relative expression level in log_2_ fold change, on the left panel. The average value of three biological replicates is reported with bars representing SD. *T*-test/Wilcoxon test *p*-value of the treatment effect is reported above each ripening stage separately. The right panel shows the RNA-seq data of the corresponding genes reporting the absolute transcript number. Figure S2. Principal component analysis (PCA) of 1000 most variable genes from 12 sequenced tomato RNA samples, extracted from control and Se-enriched fruit collected at MG and RR stages. Table S3. Description of the DEGs detected at the MG ripening stage. Table S4. Description of the DEGs detected at the RR ripening stage. Genes expressed differentially during both MG and RR stages are underlined. Table S5. The list of VOCs with quantification ion (QI), retention time (RT), retention index (RI), and weighted matching percentage. Table S6. SRM transitions and relative compound parameters for targeted polyphenol compounds (PDF)

## Notes

Acronyms and corresponding common names of genes are listed in the Supporting Information (Tables S3 and S4).

## Abbreviations

Se: selenium
NPs: nanoparticles
MG: mature green
RT: room temperature
RR: red ripe
FID: flame ionization detector
VOCs: volatile organic compounds
RI: retention index
LSD: least significant difference
PLS-DA: partial least squares discriminant analysis
GABA: γ-aminobutyric acid
PCA: principal component analysis
DEGs: differentially expressed genes
GO: Gene Ontology
FDR: false discovery rate
