# Supplementary material for: Selenium Biofortification Impacts the Tomato Fruit Metabolome and Transcriptional Profile at Ripening
Source: J Agric Food Chem. 2023 Aug 28;71(36):13554–65. doi: 10.1021/acs.jafc.3c02031 (PMC10510400; doi:10.1021/acs.jafc.3c02031)
Supplement: Supplementary file 1 — jf3c02031_si_001.pdf [file jf3c02031_si_001.pdf]

Supplementary Material

Selenium biofortification impacts tomato fruit metabolome and transcriptional profile at ripening

Anton Shiriaev[1,2*], Stefano Brizzolara[1], Carlo Sorce[3], Gaia Meoni[4], Chiara Vergata[5], Federico Martinelli[5], Elie Maza[6], Anis Djari[6], Julien Pirrello[6], Beatrice Pezzarossa[2], Fernando Malorgio[7], Pietro Tonutti[1].

[1] Crop Science Research Center, Sant'Anna School of Advanced Studies, 56127 Pisa, Italy

SB: stefano.brizzolara@santannapisa.it; PT: pietro.tonutti@santannapisa.it

[2] Research Institute on Terrestrial Ecosystems, CNR, 56124 Pisa, Italy.

AS: anton.shiriaev@iret.cnr.it; BP: beatrice.pezzarossa@cnr.it

[3] Department of Biology, University of Pisa, via L. Ghini 13, 56126 Pisa, Italy; carlo.sorce@unipi.it

[4] Magnetic Resonance Center (CERM) and Department of Chemistry "Ugo Schiff," University of Florence, 50019 Sesto Fiorentino, Italy; meoni@cerm.unifi.it

[5] Department of Biology, University of Florence, 50122 Florence, Italy

CV: chiara.vergata@unifi.it; FM: federico.martinelli@unifi.it

[6] Laboratoire de Recherche en Sciences Végétales-Génomique et Biotechnologie des Fruits-UMR5546, Université de Toulouse, CNRS, UPS, Toulouse-INP, Toulouse, France;

EM: elie.maza@toulouse-inp.fr; AD: anis.djari@toulouse-inp.fr; JP: julien.pirrello@toulouse-inp.fr

[7] Department of Agriculture, Food and Environment, University of Pisa, 56124 Pisa, Italy; fernando.malorgio@unipi.it

*Corresponding author: anton.shiriaev@iret.cnr.it

Table S1. *Gene name, GenBank number access, forward and reverse sequence of primers used in RT-qPCR for gene expression analyses.*

Gene	ID	Forward primer sequence 5’ – 3’	Reverse primer sequence 5’ -> 3’	Reference
ACO2	*Solyc12g005940.2*	ACGGGACTCGGATGTCATTA	TGCAATTGGATCACTTTCCAT	Mantelin et al. 2013, doi:10.1371/journal.pone.0063281
NOR	*Solyc10g006880.3*	AGAGAACGATGCATGGAGGTTTGT	ACTGGCTCAGGAAATTGGCAATGG	Wang et al. 2020, doi: 10.1016/j.plantsci.2020.110436
ETR3|NR	*Solyc09g075440.4*	AGAATATTGCTTTGGATGTAGC	GAGAGCACAGAGCAATAACTGCA	Shukla et al. 2017, doi: 10.1038/s41598-017-06622-0
4CL1	*Solyc03g097030.3*	TCTCAGTCGCTGCTGTAGTT	TCTTCTATGGAGTGCTTCCTC	Wu et al. 2020, doi: 10.1104/pp.20.00156
CHS1	*Solyc09g091510.3*	GGTGTGACTACCAACTCGCT	CAGAGCAAACAACAAGGACTC	Wu et al. 2020, doi: 10.1104/pp.20.00156
CHI3	*Solyc05g052240.3*	AGGCTATTGTGAATGCTCCAGTTG	TAGCACTCTCTAGCTGCACACC	This study
**Actin**	*Solyc11g005330*	CGGTGACCACTTTCCGATCT	TCCTCACCGTCAGCCATTTT	Wu et al. 2020, doi: 10.1104/pp.20.00156

Table S2. *The list of compounds identified with* [1]*D-NMR analysis, ranked based on VIP-score (Variable in Projection, weighted sum of the squared correlations between the PLS-DA components and the original variable) accordingly to the PCA analysis described on Fig. 3.*

**Compound**	**VIP**
Valerate	1.4671
Methanol	1.4647
Uridine	1.3609
Glutamine	1.2768
Citrate	1.2476
Threonine	1.2234
Formate	1.1698
Malic acid	1.1614
Alanine	1.068
Glucose	1.0642
Histidine	1.0269
GABA	1.0186
Fructofuranose	0.9813
Choline	0.9669
Trigonelline	0.9594
Phenylalanine	0.8278
AMP	0.7965
Ethanol	0.7565
2-hydroxyisovalerate	0.7504
Putrescine	0.7318
Tryptophan	0.655
Asparagine	0.652
Isoleucine	0.3923
Aspartate	0.3856
Glutamate	0.3447

Figure S1. *The expression pattern of flavonoid biosynthetic genes 4-coumarate ligase (4CL1), Chalcone synthase 1 (CHS1), Chalcone-flavanone isomerase (CHI3), and ethylene-related genes 1-aminocyclopropane-1-carboxylate oxidase 2 (ACO2), NAC domain protein (NOR), Never ripe-2 (ETR3|NR). qRT-PCR data, showing relative expression level in log2 Fold change, on the left panel. The average value of three biological replicates is reported with bars representing SD. T-test/Wilcoxon test p-value of the treatment effect is reported above each ripening stage separately. The right panel shows the RNA-seq data of the corresponding genes reporting the absolute transcript number.*

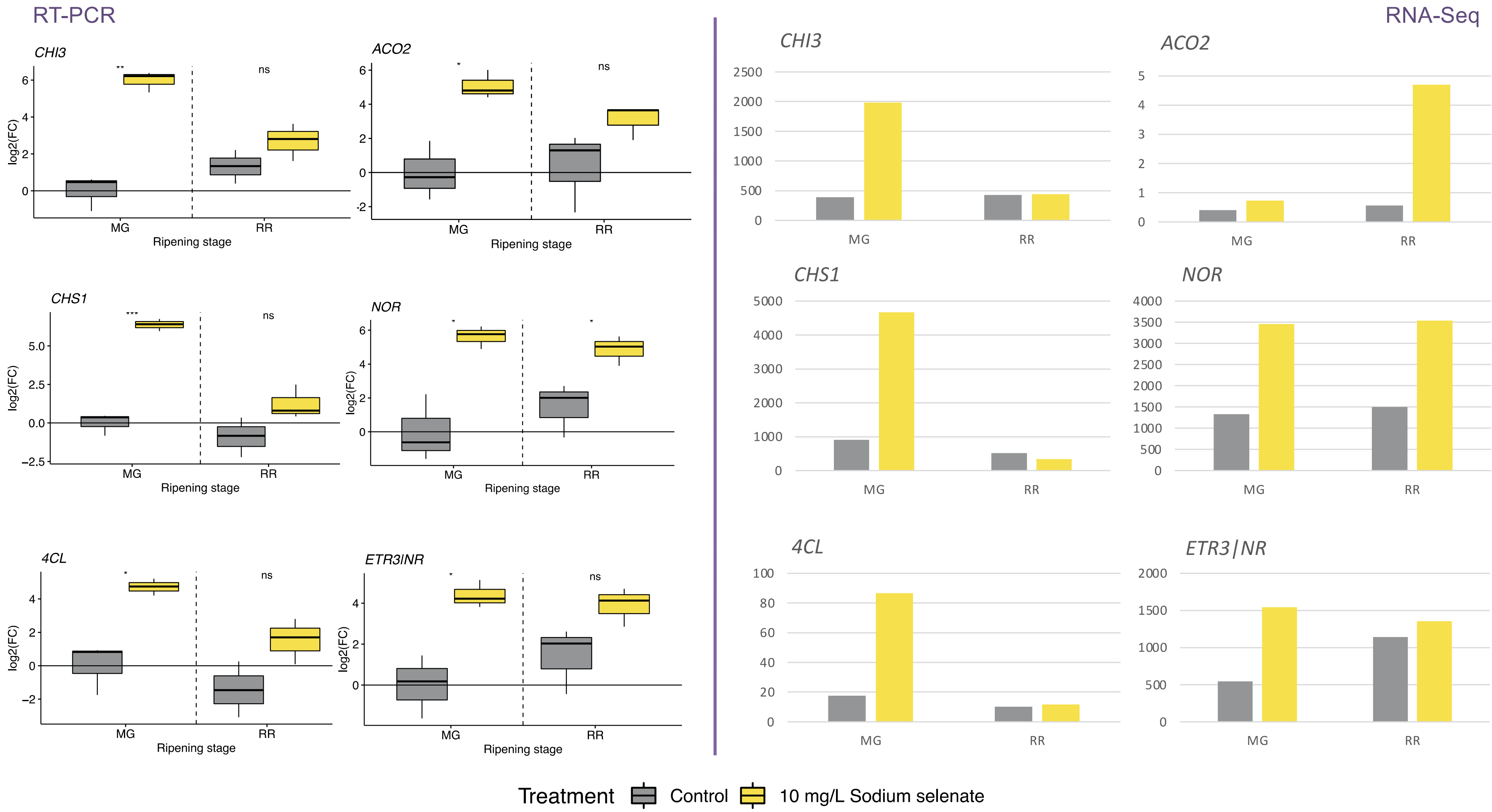

Figure S2. *Principal component analysis (PCA) of 1000 most variable genes from 12 sequenced tomato RNA samples, extracted from control and Se-enriched fruit collected at MG and RR stages.*

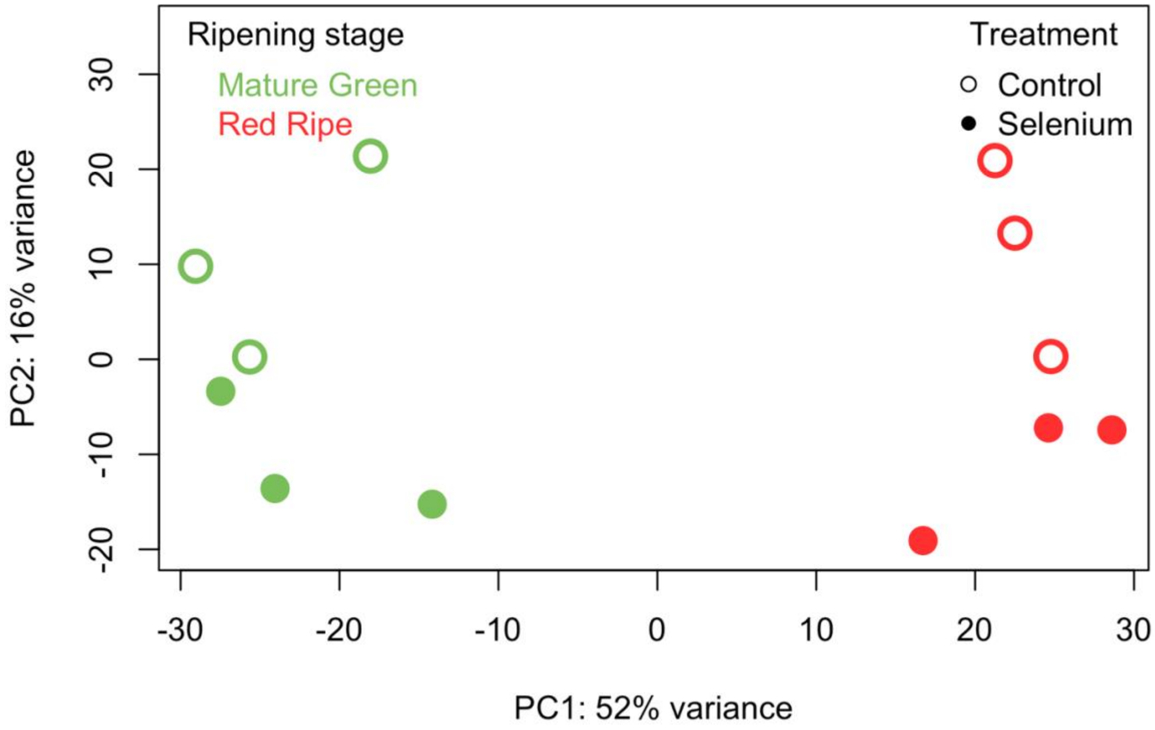

Table S3*. Description of the DEGs detected at the MG ripening stage.*

ITAG4.1	Name	Description
Solyc09g008170.3	*SAUR69*	Small auxin up-regulated RNA69
Solyc04g012140.1	*SnRK1*	SNF1-related kinase
Solyc09g091510.3	*CHS1*	Chalcone synthase 1
Solyc05g052240.3	*CHI3*	Chalcone-flavonone isomerase family protein
Solyc03g097030.3	*4CL1*	4-coumarate:CoA ligase
Solyc07g056670.3	*GA2OX1-like1*	Gibberellin 2-oxidase 2
Solyc03g044300.3	*AP2/ERF*	Ethylene-responsive transcription factor
Solyc07g008250.3	*EBF3*	F-box protein
Solyc09g089610.3	*ETR6*	Ethylene receptor-like protein
Solyc03g118190.4	*SlERF.D7*	Ethylene-responsive transcription factor
Solyc12g007070.2	*HSF*	Heat shock transcription factor
Solyc11g010710.2	*AIL1*	AP2-like ethylene-responsive transcription factor
Solyc03g096670.3	*PP2C*	Protein phosphatase 2C
Solyc09g075440.4	*ETR/NR*	Never ripe-2
Solyc12g009560.2	*EBF2*	EIN3-binding F-box protein 1
Solyc06g053710.3	*ETR4*	Ethylene receptor homolog
Solyc04g007170.3	*AP2/ERF*	Ethylene-responsive transcription factor
Solyc10g006880.3	*NOR*	NAC domain protein

Solyc05g050280.3	*GH3*	Auxin-responsive GH3 family protein
Solyc06g008030.3	*bHLH41*	BHLH transcription factor 041
Solyc10g083610.2	*CTR1*	Ethylene-inducible CTR1-like protein kinase
Solyc12g013620.2	*JA2*	Jasmonic acid 2
Solyc08g065320.3	*GRL1*	Green ripe-like 1
Solyc08g060810.3	*EBF4*	EIN3-binding F-box protein 2
Solyc03g114840.3	*MADS1*	MADS-box protein 1
Solyc03g121880.5	*NA*	NA
Solyc03g025950.4	*MSBP*	Membrane steroid-binding protein
Solyc05g056010.3	*MAIL3*	Serine/threonine-protein phosphatase 7 long form homolog
Solyc07g005500.2	*FAD6*	Omega-6 fatty acid desaturase, chloroplastic
Solyc04g008210.2	*ETAG-A3*	Xyloglucan endotransglucosylase-hydrolase
Solyc01g099600.4	*HIPP3*	Heavy metal-associated isoprenylated plant protein 3-like
Solyc02g091140.3	*SAM-METT-like10*	S-adenosyl-L-methionine-dependent methyltransferases superfamily protein
Solyc05g052040.1	*SlERF.B1*	Ripening regulated protein DDTFR10/A
Solyc03g093560.1	*SlERF.B2*	Ethylene response factor C.6
Solyc01g006950.3	*Syntaxin121-like*	Syntaxin-121-like
Solyc07g061730.3	*GA2OX2-like1*	Gibberellin 2-oxidase 5
Solyc10g086380.1	*GAI-like2*	Transcription factor GRAS
Solyc04g054690.3	*AAO*	Ascorbate oxidase
Solyc07g061720.3	*GA2OX2-like2*	Gibberellin 2-oxidase 4
Solyc04g012050.3	*SlERF.D5*	Ethylene-responsive transcription factor
Solyc07g006900.2	*SlPIN2*	Auxin efflux carrier component 2

***Table S4**. Description of the DEGs detected at the RR ripening stage. Genes expressed differentially during both MG and RR stages are underlined.*

ITAG4.1	*Name*	*Description*
Solyc12g005940.2	*ACO2*	1-aminocyclopropane-1-carboxylate oxidase 2
Solyc04g009260.3	*NA*	Disease resistance protein
Solyc11g020330.1	*HSP*	Leer-sHSP small heat shock protein
Solyc07g014620.1	*SAUR63*	Small auxin up-regulated RNA63
Solyc02g093600.3	*HSP*	Class I heat shock protein
Solyc11g067080.3	*D6PKL2*	Serine/threonine-protein kinase D6PKL2
Solyc11g069190.2	*SlARF4*	Auxin response factor 4
Solyc07g049690.3	*HPO lyase*	Hydroperoxide lyase
Solyc01g108280.3	*SlSnRK2.3*	Protein kinase
Solyc01g103430.4	*NA*	Shaggy-related protein kinase kappa

Solyc12g062250.2	*LSF1*	Phosphoglucan phosphatase LSF1, chloroplastic
Solyc02g081920.3	*SlSDG42*	Ribulose-1,5 bisphosphate carboxylase/oxygenase large subunit N-methyltransferase, chloroplastic
Solyc01g096390.4	*NPRE1-like*	DNA-directed RNA polymerase subunit
Solyc01g008960.3	*AGO4A*	Argonaute 4a
Solyc05g006590.3	*PP2A-1*	Protein phosphatase 2A catalytic subunit 1
Solyc12g094520.2	*4CLL4*	4-coumarate-CoA ligase-like 4
Solyc05g054710.3	*HEXB*	Beta-hexosaminidase
Solyc11g073050.2	*ER43*	Ethylene-responsive small GTP-binding protein
Solyc09g091030.3	*BAM7-like*	Beta-amylase
Solyc08g074550.3	*TARS*	Threonine-tRNA synthase
Solyc01g110460.4	*PPRD-2*	3-oxo-5-alpha-steroid 4-dehydrogenase family protein
Solyc07g032480.3	*SlCycT1*	CyclinT1_2
Solyc07g064470.4	*FAX-5*	Protein FATTY ACID EXPORT 5
Solyc10g074710.2	*NA*	Serine/threonine-protein kinase
Solyc01g009310.3	*DWARF5-like1*	Meloidogyne-induced giant cell protein DB217
Solyc03g007310.3	*PYL8*	Abscisic acid receptor PYL8
Solyc11g007580.3	*SlDML3*	DNA demethylase 3
Solyc10g047140.2	*NA*	Receptor-like kinase
Solyc11g069810.3	*NA*	Cysteine proteinases superfamily protein
Solyc01g090430.3	*NRC1*	Natural cytotoxicity triggering receptor 1
Solyc03g122350.3	*CYP450*	Cytochrome P450
Solyc06g008870.2	*GID1B-like*	Gibberellin receptor
Solyc10g083970.1	*SAMS*	S-adenosylmethionine synthase
Solyc07g049550.3	*ACO3*	1-aminocyclopropane-1-carboxylate oxidase 2
Solyc12g094660.2	*NA*	Disease resistance protein
Solyc12g056980.1	*ERF.H11*	Ethylene-responsive transcription factor
Solyc07g054220.1	*ERF*	Ethylene-responsive transcription factor
Solyc06g069580.3	*HPSE-like 1*	Heparanase-like protein 1
Solyc01g006950.3	*Syntaxin121-like*	Syntaxin-121-like
Solyc07g061730.3	*GA2OX2-like1*	Gibberellin 2-oxidase 5
Solyc11g020230.1	*CCR4*	Serine/threonine-protein kinase-like protein CCR4
Solyc06g076020.3	*HSP*	Heat shock protein 70 kD
Solyc02g092820.4	*SlGH3_4*	IAA-amido synthetase 3-4
Solyc12g096900.2	*NA*	Disease resistance protein family
Solyc07g008630.1	*EIX2*	Ethylene-inducing xylanase receptor 2
Solyc07g063850.3	*SlGH3_9*	IAA-amido synthetase 3-9
Solyc08g006740.3	*AADC2*	Aromatic amino acid decarboxylase 2
Solyc01g087780.2	*SBT4A*	Serine protease SBT4A

Table S5. *The list of VOCs with quantification ion (QI), retention time (RT), retention index (RI) and weighted matching percentage.*

Compound	QI	RT, min	RI	Weighted match, %
1-penten-3-one	55	8.577	1060.6	98
Hexanal	41	9.991	1115.5	96
1-butanol-3-methyl	41	12.684	1217.8	89
2-Hexenal	41	13.841	1257.5	99
3-Hexenal, (Z)-	41	14.953	1295.6	87
2-Heptenal, (E) -	41	17.012	1363	95
5-Hepten-2-one, 6-methyl	43	17.14	1367	95
3-Hexen-1-ol, (Z)-	41	18.162	1401.1	92
Nonanal	41	18.899	1425.1	96
Sorbaldehyde	81	19.26	1435.2	83
2-Izobuthylthiazol	99	19.494	1442.7	96
1-Octen-3-ol	57	19.989	1459.2	91
2-Octenal, (E) -	70	20.242	1467.2	90
dl-6-Methyl-5-hepten-2-ol	95	20.4	1472.4	98
2,4-Heptadienal, (E,E)-	81	21.265	1500.5	91
Decanal	43	22.113	1528.5	91
Linalool	71	23	1557.7	94
1-Octanol	43	23.32	1568.2	96
Benzaldehyde	77	23.55	1576	97
PTAL	119	27.37	1705.2	98
3-cyclohexene-1-methanol, α, α, 4-trimethyl	59	28	1727.5	90
Citral	69	29.19	1770	96
Salicylic acid isopropyl ester	120	31.04	1837.2	99
Damascenone	69	31.93	1870.1	88
Hexanoic acid	60	32.1	1876.2	93
Geranyl acetone	43	32.32	1884.9	97
Phenylethyl alcohol	91	34.09	1953.6	98
β-Ionone	177	35.05	1992	99
β-Ionone epoxide	123	36.5	2049.3	95

Table S6. *SRM transitions and relative compound parameters for targeted polyphenol compounds.*

Compound	*Acronym*	*MW*	*Q1*	*Q3*	*DP(V)*	*CE(eV)*	*CXP(V)*	*RT (min)*
4-Coumaric acid	PCA	164	163	119	-65	-18	-11	3.35
Trans-Ferulic acid	TFRA	194	193	134	-62	-20	-8	3.65
Resveratrol	RSV	228	227.1	185	-179	-25	-9	4.46
Naringenin	NRG	272	270.9	150.9	-120	-25	-10.5	5.13
Phloretin	PHL	274	273	167	-103	-38	-11	5.04
Apigenin	APG	270	268.9	117	-120	-49	-14	5.02
Luteolin	LTO	286	284.9	133	-130	-44.6	-17.4	4.6
Catechin	CTC	290	289	244.9	-108	-22	-11	2.69
Epicatechin	ECTC	290	289	244.9	-108	-22	-11	2.98
Quercetin	QCT	302	301	150.9	-113	-38	-8	4.64
Chlorogenic acid	CGA	354	353	191	-61	-24	-9	2.56
Piceid	PCD	390	389.1	227	-125	-32	-11	3.54
Phloridzin	PDZ	436	435.1	272.9	-135	-23	-5	4.01
Kampferol-3-O-glucoside	KPF3G	448	447.1	284.1	-202	-39	-11	3.69
Quercetin-3-O-glucoside	QCT3G	464	463.1	300	-154	-37	-5	3.46
Kampferol-3-O-rutinoside	KPF3R	594	593.2	284.9	-138	-40	-5	3.52
Rutin	RTN	610	609.2	299.9	-154	-48	-11	3.29
